# EPRS1 correlates with malignant progression in hepatocellular carcinoma

**DOI:** 10.1186/s13027-023-00503-0

**Published:** 2023-05-03

**Authors:** Chen Yang, Xiaofeng Yang, Chenghao Liu, Jun Hou, Xueling Chen, Lianghai Wang, Xiangwei Wu

**Affiliations:** 1 NHC Key Laboratory of Prevention and Treatment of Central Asia High Incidence Diseases, the First Affiliated Hospital, Shihezi University School of Medicine, Shihezi, Xinjiang China; 2 Key Laboratory of Xinjiang Endemic and Ethnic Diseases, Shihezi University School of Medicine, Shihezi, Xinjiang China; 3 Department of Oncology, Lianyungang Oriental Hospital, Lianyungang, Jiangsu China; 4 Department of Pathology, the First Affiliated Hospital, Shihezi University School of Medicine, Shihezi, Xinjiang China; 5 Department of Hepatobiliary Surgery, the First Affiliated Hospital, Shihezi University School of Medicine, Shihezi, Xinjiang China

**Keywords:** Glutamyl-prolyl-tRNA synthetase 1, HCC, Prognosis, Proteomics, Amplification.

## Abstract

**Background:**

Glutamyl-prolyl-tRNA synthetase 1 (EPRS1) is an aminoacyl-tRNA synthase involved in the pathology of cancer and other diseases. In this study, we investigated the carcinogenic function, potential mechanism, and clinical significance of EPRS1 in human hepatocellular carcinoma (HCC).

**Methods:**

The expression, clinical significance, and prognostic value of EPRS1 in HCC were assessed using the TCGA and GEO databases. The function of EPRS1 in HCC cells was detected by CCK-8, Transwell, and hepatosphere formation assays. Immunohistochemistry was used to explore the difference in EPRS1 levels in HCC tissues and peri-cancerous tissues. The mechanism of EPRS1 was studied using a proteomics method. Finally, cBioportal and MEXEPRSS were used to analyze the variations involved in the differential expression of *EPRS1*.

**Results:**

EPRS1 was frequently upregulated at the mRNA and protein levels in liver cancer. Increased EPRS1 correlated with shortened patient survival. EPRS1 could promote cancer cell proliferation, characteristics of cell stemness, and mobility. Mechanistically, EPRS1 played a carcinogenic role by upregulating several downstream proline-rich proteins, primarily LAMC1 and CCNB1. In addition, copy number variation could contribute to the high expression of *EPRS1* in liver cancer.

**Conclusion:**

Together, our data imply that enhanced EPRS1 contributes to the development of HCC by increasing the expression of oncogenes in the tumor microenvironment. EPRS1 may be a successful treatment target.

**Supplementary Information:**

The online version contains supplementary material available at 10.1186/s13027-023-00503-0.

## Background

Liver cancer is one of the most prevalent kinds of cancer and one of the most common causes of cancer-related mortality. Its prognosis varies based on the etiology, geographical location, disease stage at the time of diagnosis, and other factors [1]. Alcohol misuse, chronic hepatitis B or C virus infection, and several metabolic illnesses are factors that contribute to the development of hepatocellular carcinoma (HCC) [2]. However, the specific molecular mechanism of HCC pathogenesis is still poorly understood [3]. Therefore, it is of great significance to explore novel tumor regulatory factors and potential mechanisms of tumor progression to provide novel biomarkers and therapeutic targets for HCC.

Protein synthesis is the process of translating base sequences presented in messenger RNA (mRNA) molecules into proteins or polypeptide chains. Before synthesizing polypeptide chains, amino acids must be bound with their specific tRNA to the corresponding position of mRNA. Aminoacyl-tRNA synthetase (ARS) is responsible for catalyzing the binding between certain amino acids and its associated tRNA to produce diverse aminoacyl-tRNA [4, 5]. In humans, 36 ARS genes encode cytoplasmic and mitochondrial enzymes, of which 16 operate exclusively in the cytoplasm, 17 in the mitochondria, and three being bifunctional [6]. Pathogenic variations in ARSs can cause various illnesses, such as cancer, fibrosis, and autoimmune diseases, which exhibit significant clinical diversity in a wide range of organs without apparent logical progression [7–9]. Therefore, ARS has gathered wide research interest, and their prognostic value and regulatory mechanism in tumors deserve further study.

In this study, we screened the expression profile of cytosolic ARSs to find their association with the progression of liver cancer. Data from bioinformatics analyses, HCC cells, and clinical specimens were combined to describe the novel mechanisms by which glutamyl-prolyl-tRNA synthetase 1 (EPRS1) promotes the synthesis of several downstream proteins and the progression of liver cancer.

## Methods

### Cell culture and transfection

Human HCC cell lines SNU-387, HepG2, Huh7, and Hep3B were obtained from the Cell Bank, Type Culture Collection, Chinese Academy of Science. Huh7 cells were grown in DMEM supplemented with 10% fetal bovine serum (FBS). SNU387 cells were cultured in RPMI1640 medium supplemented with 10% FBS. HepG2 and Hep3B cells were grown in MEM supplemented with 10% FBS. Halofuginone (S8144) was purchased from Selleck. The use of mycoplasma-free cells was ensured throughout all experiments.

### Cell transfection

Small interfering RNAs (siRNAs) targeting *EPRS1* (siEPRS1#1 sense was GGAUGAUACUCCUGCUGAATT, antisense was UUCAGCAGGAGUAUCAUCCTT; siEPRS1#2 sense was CCUGACAACUCGAACUAUUTT, antisense was AAUAGUUCGAGUUGUCAGGTT) were ordered from GenePharma (Shanghai, China). Plasmid overexpressing *EPRS1* (NM_004446) based on the GV219 vector was purchased from GeneChem. Lipofectamine 2000 and Lipofectamine 3000 (Invitrogen) transfection reagents were used to transfect siRNAs and plasmids following the manufacturer’s recommendations.

### Real-time PCR

Total RNA was extracted using the E.N.Z.A. Total RNA Kit I (R6834, OMEGA) according to the manufacturer’s instructions. cDNA was synthesized from the extracted total RNA using the RevertAid First Strand cDNA Synthesis Kit (Thermo Scientific). The following primers were used to quantify gene expression using a CFX96 Touch Real-Time PCR Detection System (Bio-Rad): *EPRS1* forward, ATCTTCTTCCTTTCGCGGGG; *EPRS1* reverse, CAGCAAAGCTCCTAGCGGA; *ACTB* forward, CATGTACGTTGCTATCCAGGC; *ACTB* reverse, CTCCTTAATGTCACGCACGAT. *EPRS1* expression was quantified in relation to *ACTB* expression using the 2^−ΔΔCt^ method.

### Cell proliferation assay

Cells were seeded at 5 × 10^3^ cells/well in 96-well plates with transparent bottoms. CCK-8 (Dojindo) was used to measure cell proliferation of different wells at the indicated time points, following the manufacturer’s recommendations. Varioskan LUX multimode microplate reader (Thermo Scientific) was used to measure the absorbance at OD_450nm_.

### Hepatosphere formation assay

A total of 2 × 10^3^ cells/well were grown on 24-well ultra-low adherence plates using single-cell suspensions. Serum-free DMEM/F12 media with 20 ng/mL EGF, 20 ng/mL fibroblast growth factor, and 2% B27 were used for the propagation of the seeded cells. The development of hepatospheres was seen under the microscope after 10 days of culture, and both the number of hepatospheres and their size were determined.

### Cell migration assay

Using Transwell cell culture inserts with 8.0-µm pores, a cell migration test was conducted in 24-well plates (Corning). Hep3B, Huh7, and HepG2 cells were seeded at 5 × 10^4^ cells/well in the top chamber with serum-free DMEM. The lower chamber contained DMEM with 10% FBS. These cells were permitted to migrate for 48 h. The cells on the inner surface of the inserts were removed using cotton swabs. The inserts were fixed with 4% formaldehyde at 4 °C for 15 min, and the cells situated on the bottom surface of the inserts were stained for 20 min with 0.1% crystal violet before being counted under a light microscope in three randomly selected areas.

### Mass spectrometry and data processing

The protein mixtures from sample tissues were extracted, processed, and digested. Briefly, peptide samples are labeled with TMT reagents, and the peptide mixtures were graded using an Agilent 300 Extend C18 column with high-performance liquid chromatography and then evaluated using an Orbitrap Exploris 480 mass spectrometry system (Thermo Fisher). To examine all control and suppressed EPRS1 raw files, we used Proteome Discoverer (v2.4.1.15) to search for proteins in the homo sapiens 9606 SP 20,201,214.fasta database (20,395 sequences) and added them to the reverse database. While the false positive rate resulting from random matching was computed, a co-contamination database was included to reduce the impact of contaminating proteins on those identified.

### Western blot

Equal amounts of cell lysates were separated by sodium dodecyl sulfate-polyacrylamide gel electrophoresis and transferred to polyvinylidene difluoride membranes before immunoprobed with specific antibodies against EPRS1 (HPA026490; Sigma-Aldrich), LAMC1 (sc-13,144; SANTA CRUZ Biotechnology), and β-actin (#3700; Cell Signaling Technology). Immobilon Western Chemiluminescent HRP Substrate (WBKLS0050; Millipore) was used to detect the signals by capturing images with the Clinx ChemiScope Chemiluminescence imaging system.

### Human specimens

A total of 107 primary HCC specimens and 95 peri-tumoral tissues (84 matched pairs) were obtained from patients undergoing surgical excision without prior radio- or chemotherapy at the First Affiliated Hospital, Shihezi University School of Medicine. Informed consent was obtained from patients upon research authorization by the Ethics Committee of the First Affiliated Hospital of Shihezi University School of Medicine.

### Immunohistochemistry

Antigen retrieval was performed on paraffin slices after they had been deparaffinized and dehydrated. To eliminate non-specific background staining, sections were blocked with 5% goat serum for 1 h before being treated with the primary antibody to EPRS1 (HPA026490; Sigma-Aldrich) at 4 °C overnight. Thereafter, the sections were washed with TBST and treated with 3% H_2_O_2_ to inhibit endogenous peroxidase activity. Afterward, the samples were treated with a secondary antibody coupled to horseradish peroxidase and detected using a DAB kit (ZLI-9018; ZSGB Bio, Beijing, China) before being analyzed. The degree of immunostaining in each sample was determined for each sample according to the level of staining (intensity score: 0, no staining; 1, weak staining; 2, moderate staining; 3, strong staining) and the percentage of positive cells (extent score: 0, ≤ 5%; 1, 6–25%; 2, 26–50%; 3, 51–75%; 4, 76–100%). The final immunoreactivity score of EPRS1 levels was the product of the intensity score and extent score for each case.

### Statistical analysis

Data are presented as mean ± standard error of the mean. GraphPad Prism v8 was used to conduct statistical analyses. The Mann-Whitney test, Wilcoxon matched-pairs signed-rank test, two-tailed Student’s *t*-test, or ANOVAs with post-hoc testing were used to compare the groups. Survival estimates were obtained by using Kaplan-Meier analysis and the log-rank test. The significance of correlation between genes was evaluated by Spearman’s correlation. *P*-value < 0.05 was considered statistically significant: *P < 0.05, **P < 0.01, ***P < 0.001.

## Results

### EPRS1 is upregulated in liver cancer and correlates with poor prognosis

**Fig. 1:**
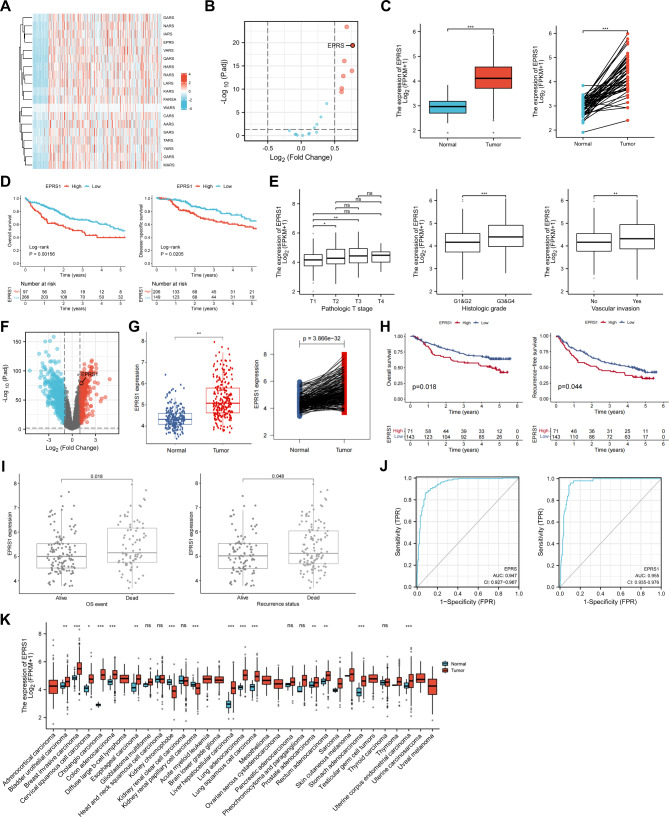
EPRS1 was upregulated in liver cancer and correlated with poor prognosis. A, Expression of 19 cytosolic ARSs in TCGA-LIHC cohort. B, Volcanic map of 19 differentially expressed cytosolic ARSes. C, *EPRS1* mRNA levels upregulated in tumor samples from TCGA-LIHC. D, Kaplan-Meier curves for five-year overall survival (left) and disease-specific survival (right) according to *EPRS1* expression of patients with liver cancer from the TCGA-LIHC dataset. E, Association between *EPRS1* expression and clinicopathological factors in liver cancer. F, Volcano map of *EPRS1* expression in GSE14520 (GPL3921) dataset. G, *EPRS1* expression in 225 hepatitis B virus-related HCC and 220 non-tumor tissues (left), or 213 paired tumor and non-tumor tissues from the GSE14520 dataset. H, Kaplan-Meier curves for overall survival (left) and recurrence-free survival (right) of patients with liver cancer from the GSE14520 dataset according to *EPRS1* expression. I, Correlation between *EPRS1* expression and clinicopathological factors in liver cancer from the GSE14520 dataset. J, ROC analysis indicated that *EPRS1* mRNA levels were specific and sensitive biomarkers for HCC detection in both TCGA-LIHC (left) and GSE14520 (right) datasets. K, High *EPRS1* expression in other tumors in the TCGA database

We evaluated the mRNA levels of 19 cytosolic aminoacyl-tRNA synthetases (16 operate exclusively in the cytoplasm and three both in the cytoplasm and mitochondrial) in tumor samples and normal tissues based on The Cancer Genome Atlas Liver Hepatocellular Carcinoma (TCGA-LIHC) project. The results indicated the differential expression of *EPRS1* was most obvious in the tumor samples compared with normal tissues (Fig. 1A–C). To explore the impact of *EPRS1* expression on the prognosis of patients with HCC, survival analysis was conducted. The Kaplan-Meier curves indicated that the five-year overall survival and disease-specific survival of patients with high *EPRS1* expression were significantly shortened (Fig. 1D). Furthermore, increased *EPRS1* expression was shown to be substantially associated with advanced pathologic T stage, higher histologic grade, and the presence of vascular invasion (Fig. 1E). To confirm the reliability of the bioinformatics analysis results, we performed the same analysis using liver cancer data from the GSE14520 cohort, and the results were consistent with the previous findings (Fig. 1F–I). The receiver operating characteristic (ROC) curve analysis further indicated that *EPRS1* expression was highly sensitive and could serve as specific biomarkers to distinguish HCC from normal liver tissues in both datasets (Fig. 1J). In addition, by analyzing the expression of *EPRS1* in all the cancer types from the TCGA database, we found that *EPRS1* is highly expressed in a variety of other tumors (Fig. 1K).

### Knockdown of EPRS1 depressed malignant phenotypes

**Fig. 2:**
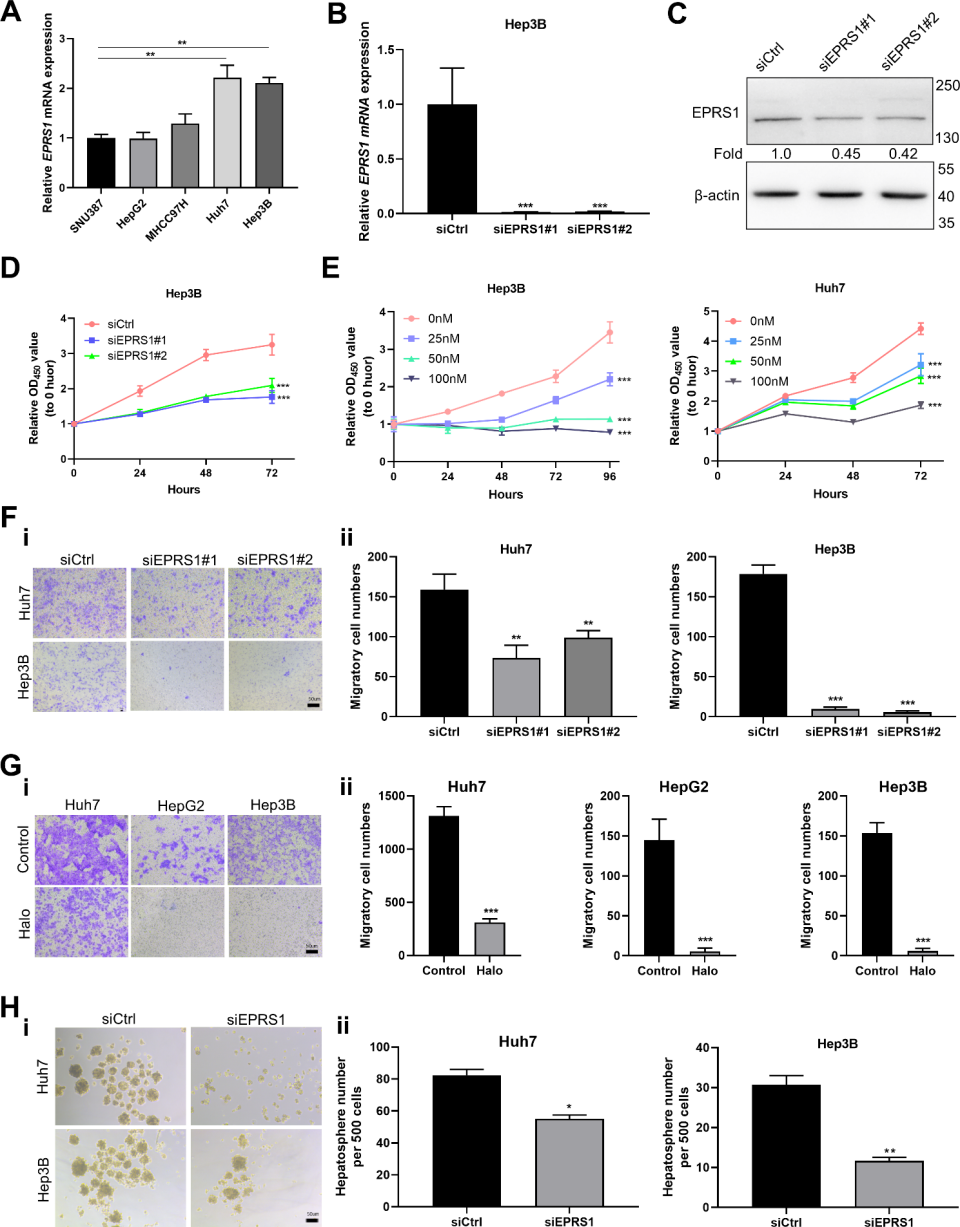
*EPRS1* knockdown depressed malignant phenotypes. A, Detection of relative *EPRS1* mRNA expression in five HCC cell lines. The cell line with the lowest *EPRS1* mRNA level was set to 1. B, siRNAs were employed to knock down *EPRS1* expression. *EPRS1* downregulation confirmed at mRNA level in Hep3B cells. C, Validation of EPRS1 downregulation at the protein level by Western blot. Predicted EPRS1 molecular mass: 170.6 kDa. D, Proliferation ability of Hep3B cells after siRNA knockdown of *EPRS1* expression was assessed at the indicated time points (n ≥ 6 for each time point) by CCK8 assay. E, Growth curves of Hep3B and Huh7 cells under halofuginone treatment measured by CCK-8 assay. F, Transwell migration assay on cells treated with siRNA targeting *EPRS1*. F-i, Representative images of membranes with violet spots indicating migrated cells stained with crystal violet. F-ii, Statistical analysis of migratory Huh7 (left) and Hep3B (right) cell numbers. G, Migration assay on cells treated with 100 nmol/L halofuginone (Halo) for 48 h. G-i, Representative images of migrated cell spots. G-ii, Statistical analysis of migratory Huh7 (left), HepG2 (middle), and Hep3B (right) cell numbers. H, Hepatosphere formation assay on cells treated with siRNA targeting *EPRS1*. H-i, Representative images of hepatospheres. H-ii, Quantification of hepatospheres formed by Huh7 (left) and Hep3B (right) cells after *EPRS1* silencing

Detection of *EPRS1* expression of five HCC cells showed high expression in Huh7 and Hep3B cells, while low in SNU387 cells (Fig. 2A). To investigate whether *EPRS1* expression levels would affect the malignancy of HCC cells, we employed siRNAs to knockdown *EPRS1* in Hep3B and Huh7 cells. Real-time PCR and Western blot were used to confirm that the gene deletion was successful (Fig. 2B–C). Halofuginone, an EPRS1 competitive inhibitor, was also used to inhibit its biological functions. We found that depletion of *EPRS1*, by either knockdown or functional suppression, dramatically suppressed HCC cell proliferation and cell migration ability (Fig. 2D–G). Moreover, knockdown of *EPRS1* substantially inhibited the hepatosphere-formation ability of HCC cells (Fig. 2H). These results indicated that *EPRS1* could drive cell proliferation, mobility, and the characteristics of cell stemness in HCC.

### Overexpression of EPRS1 promoted cell proliferation and migration

**Fig. 3:**
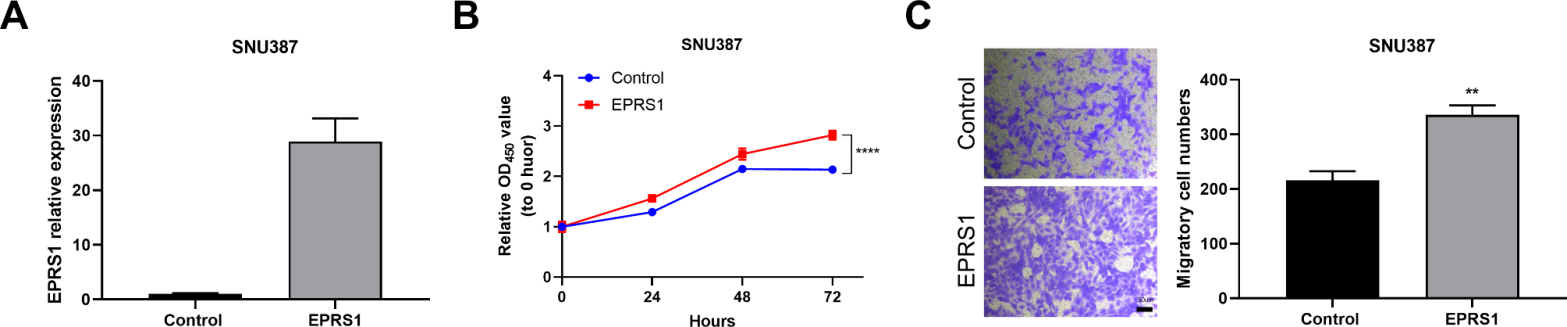
EPRS1 overexpression significantly promoted cell growth and motility. A, Real-time PCR analysis confirming elevated EPRS1 level in SNU387 cells after ectopic expression of *EPRS1*. B, Growth curves of SNU387 cells with *EPRS1* overexpression assessed by CCK-8 assay. C, Representative images (left) and quantification (right) of the *EPRS1*-overexpressing cells that migrated through the Transwell membrane

To consolidate our findings, we further overexpressed *EPRS1* in SNU387 cells (Fig. 3A). Cell proliferation was dramatically increased when *EPRS1* was ectopically expressed in SNU387 cells as opposed to the vector control (Fig. 3B). The Transwell assay also revealed that overexpression of *EPRS1* increased cell migration (Fig. 3C). These results suggest that the increased expression of *EPRS1* can enhance the proliferation and migration of HCC cells.

### EPRS1 modulated proline-rich protein expression

**Fig. 4:**
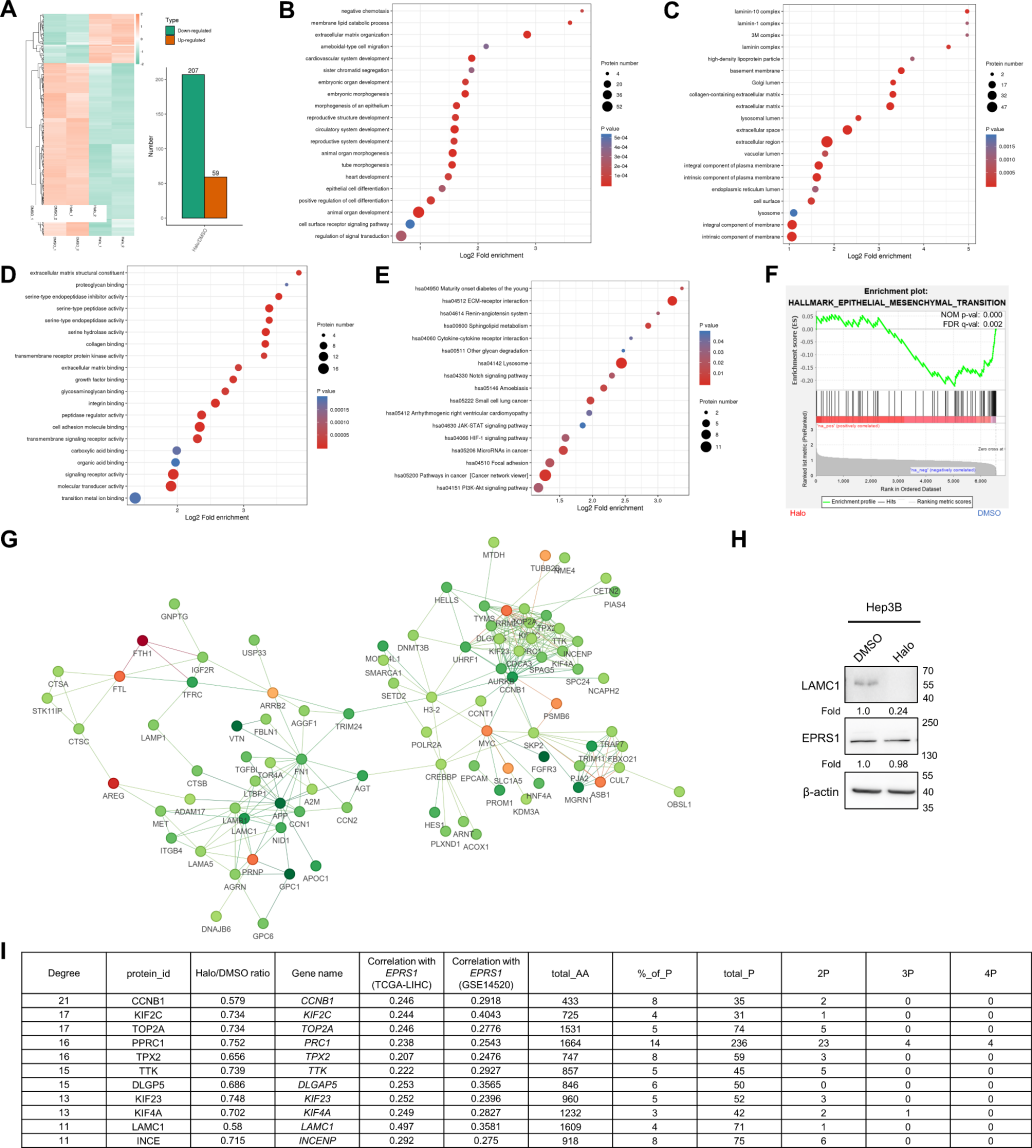
EPRS1 modulated proline-rich protein expression. A, Heat map of abundance changes in cells treated with 300 nmol/L halofuginone for 24 h compared to vehicle control (average of *n* = 2). Red represents upregulated proteins and green represents downregulated proteins. B–D, Gene Ontology enrichment of downregulated proteins, including biological process (B), cellular component (C), and molecular function (D). E, KEGG pathway enrichment of downregulated proteins. F, Gene set enrichment analysis showing significant enrichment of the indicated gene set comparing the inhibitor group with the DMSO control group. FDR, false discovery rate. G, Differential protein interaction analysis using the STRING database. Different colors represent the differential expression of proteins (red, upregulated proteins; green, downregulated proteins). The top 50 proteins with the closest interaction were screened and mapped in a protein interaction network. H, LAMC1 level in Hep3B cells treated with 300 nmol/L halofuginone for 24 h was determined by Western blot. I, Correlation between *EPRS1* expression and downregulated proteins represented by LAMC1 and CCNB1 in TCGA-LIHC and GSE14520 datasets. P, proline

To gain a complete understanding of the effects of EPRS1 on HCC progression, we performed a proteomic analysis on Hep3B cells treated for 24 h with either 300 nmol/L halofuginone or DMSO control. We discovered more than 66,000 proteins using a mass spectrometry-based approach to quantify the protein samples. We found that halofuginone had a substantial effect on the levels of 266 proteins (4.03% of the total) compared to the DMSO control. The bulk of differentially regulated proteins was downregulated (207 downregulated vs. 59 upregulated) (Fig. 4A and Supplementary Table 1 ). To determine whether differentially expressed proteins had a significant enrichment trend in functional pathways, we conducted enrichment analyses of the downregulated proteins based on Gene Ontology classification. Results illustrated that downregulated proteins were mainly involved in the extracellular matrix organization, laminin complex, collagen-containing extracellular matrix, extracellular matrix structural constituent, and cell adhesion molecule binding (Fig. 4B–D and Supplementary Table 2 ). In addition, we found out that downregulated proteins are mostly enriched in ECM-receptor interaction, pathways in cancer, and focal adhesion by KEGG pathway enrichment analysis (Fig. 4E and Supplementary Table 3 ). Gene set enrichment analysis was performed to further validate the possible roles of EPRS1 in the progress of liver cancer. It was found that gene sets associated with metastasis (hallmark epithelial-mesenchymal transition) were considerably overrepresented in the DMSO control group rather than the halofuginone treatment group (Fig. 4F). The differential proteins with 1.3 fold-change were compared with the protein network interaction database of STRING (v11.0) to acquire the interaction connections of the differentially expressed proteins. We screened the top 50 proteins with the closest relationships and displayed the protein interaction network (Fig. 4G). Noteworthy, LAMC1 and CCNB1 were particularly downregulated (Fig. 4I and Supplementary Table 1 ). Western blot confirmed that halofuginone treatment downregulated the LAMC1 level in Hep3B cells (Fig. 4H). In addition, positive correlations between the expression of *EPRS1* with proline codon-rich *LAMC1* and *CCNB1* were also observed in both the TCGA-LIHC and GSE14520 datasets (Fig. 4I). As a consequence of these findings, the oncogenic impact of EPRS1 may be generated by promoting with the production of downstream proteins, primarily LAMC1 and CCNB1.

### Copy number variation causes high expression of EPRS1 in liver cancer

**Fig. 5:**
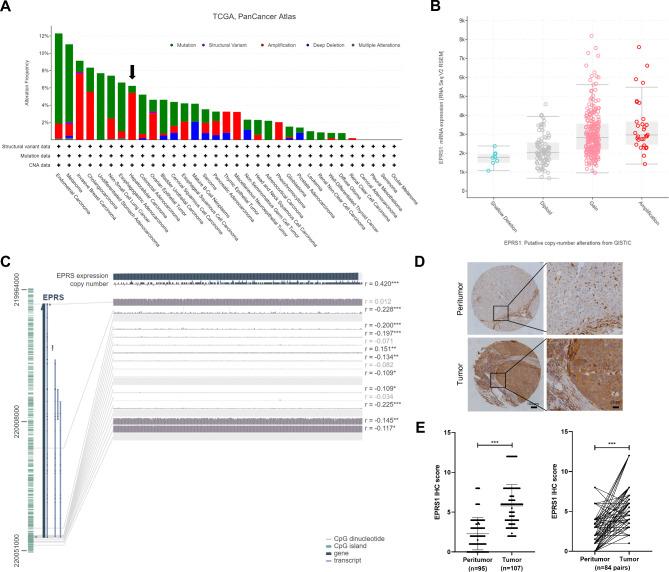
High *EPRS1* expression positively correlated with copy number in liver cancer. A, Analysis of copy number variation in *EPRS1* for various tumors in TCGA data sets using the public cBioPortal site. B, *EPRS1* mRNA expression in HCC with the indicated copy-number alterations derived from the cBioPortal for Cancer Genomics. C, The correlation between *EPRS1* expression, copy number, and DNA methylation in liver cancer was visualized using the MEXPRESS tool. D, Representative immunohistochemical staining of EPRS1 level in liver cancer and peri-tumor tissues. E, Statistical analysis of EPRS1 immunohistochemistry scores in all tumor (n = 107) and peri-tumor tissues (n = 95; left) or paired tumor and peri-tumor tissues (n = 84 pairs; right)

Among the variations in the DNA sequence found in the human genome, copy number variation (CNV) is a variable that may alter the expression of local and distant genes, resulting in phenotypic differences. To figure out whether the high expression of *EPRS1* in liver cancer is related to CNV, we used the cBioPortal online tool and found that *EPRS1* has obvious CNV in HCC and other tumors (Fig. 5A–B). Furthermore, MEXEPRSS online analysis revealed a strong connection between *EPRS1* expression and its copy number in HCC (*R* = 0.419; Fig. 5C). To confirm the clinical significance of elevated *EPRS1* expression in liver cancer, we examined the protein levels of EPRS1 on a tissue microarray using immunohistochemical staining (Fig. 5D). The results revealed stronger EPRS1 staining in tumor regions than their matched non-tumor tissues (Fig. 5E).

## Discussion

To date, only a few studies have reported on the association between *EPRS1* expression and the prognosis of patients with tumors. In ER^+^ breast cancer, high expression of *EPRS1* predicted shorter overall survival [10]. The PCG-lncRNA signature, which included *EPRS1*, could be used to separate patients with glioblastoma into two groups with dramatically differing survival rates (i.e., high- and low-risk groups) [11]. Moreover, EPRS1 was also described as a marker for gastric cancer presenting with a greater cell proliferation and tumor growth phenotype [12]. Our results showed that *EPRS1* expression was upregulated at both the mRNA and protein levels in liver cancer. Patients with high expression of *EPRS1* had shorter overall and disease-free survival. In addition, we found that inhibition of EPRS1 effectively decreased cancer cell proliferation and migration, whereas enforced EPRS1 expression promoted malignancy, indicating that EPRS1 might contribute to tumor development. Therefore, we have shown for the first time that EPRS1 is a potential predictive biomarker of prognosis in patients with HCC.

Recently, EPRS1 was reported to increase the translation efficiency of proline-rich pro-fibrotic protein during cardiac fibrosis [13]. In this study, public data sets and subsequent experimental analysis showed that inhibition of *EPRS1* expression downregulated several proline-rich proteins, among which LAMC1 and CCNB1 were most affected. Laminin is a trimer compound composed of α, β, and γ chains. LAMC1 specifically encodes the laminin γ1 chain and is significantly expressed in the basement membrane. It is an important regulator of the extracellular matrix and has certain effects on cell differentiation, migration, and adhesion [14]. LAMC1 is also involved in a variety of biological and pathological processes including tumor cell development, invasion, and metastasis [15, 16]. As a pretext to invasion, tumor cells must first adhere to the surrounding tissue where laminin plays a crucial part. Then the stroma must be broken down before infiltrating cells are allowed to colonize newly created intracellular niches [17]. Previous studies have found that LAMC1 plays a role in the progression of gastric cancer [18], endometrial cancer [19], prostate cancer [20], breast cancer [21], and other tumors. In HCC, LAMC1 could promote malignancy by regulating the expression of *PKM2* via the AKT pathway, competing for microRNA-124 binding with CD151, or other pathways [22–24]. Therefore, we hypothesized that EPRS1 plays a carcinogenic role by promoting the expression of downstream oncogenic factors such as *LAMC1*.

CNVs can effectively affect the expression of genes related to the progression of HCC [25]. Using the TCGA-LIHC dataset, we discovered considerable duplication of the *EPRS1* region in liver cancer tissues, suggesting the mechanism of EPRS1 upregulation in liver cancer. However, further research on this particular process is required.

In conclusion, our study highlights that the CNV of *EPRS1* increased *EPRS1* expression, which promoted protein synthesis of downstream oncogenic genes and promoted liver cancer progression. Therefore, targeting EPRS1 may be a new therapeutic strategy for patients with HCC.

## Electronic supplementary material

Below is the link to the electronic supplementary material.

Supplementary Material 1

## Data Availability

The data sets analysed during this study are available in public, open access repositories listed in this article.

## List of abbreviations

ARS: aminoacyl-tRNA synthetase
CNV: copy number variation
EPRS1: glutamyl-prolyl-tRNA synthetase 1
FBS: fetal bovine serum
HCC: hepatocellular carcinoma
mRNA: messenger RNA
ROC: receiver operating characteristic
TCGA-LIHC: The Cancer Genome Atlas Liver Hepatocellular Carcinoma
